# Risk for Donor-Derived Syphilis after Kidney Transplantation, China, 2007–2022

**DOI:** 10.3201/eid3007.240009

**Published:** 2024-07

**Authors:** Saifu Yin, Lijuan Wu, Congke Liu, Zihao Jia, Jiapei Wu, Fan Zhang, Xianding Wang, Turun Song, Tao Lin

**Affiliations:** West China Hospital, Sichuan University, Chengdu, China

**Keywords:** Syphilis, donor-derived, organ transplantation, Treponema pallidum, bacteria, China

## Abstract

To evaluate the risk of acquiring syphilis from a donated kidney, we evaluated kidney transplantation pairs from West China Hospital, Sichuan, China, during 2007–2022. Donor-derived syphilis was rare. Risk may be higher if donors have active syphilis and may be reduced if recipients receive ceftriaxone.

Syphilis transmission remains a public health challenge; ≈7.1 million new cases were reported in 2020 (*1*). Despite its relatively low incidence, if left untreated, syphilis results in substantial disease and death. Although the causative organism of syphilis, *Treponema pallidum*, is primarily transmitted sexually and vertically, transmission through solid organ transplantation is theoretically possible (*2*).

Kidneys are the most commonly and successfully transplanted solid organs. According to the Chinese National Renal Data System (http://www.cnrds.net), in 2022, 177,445 persons began dialysis, bringing the total undergoing dialysis to 984,809. However, according to the China Scientific Registry of Kidney Transplantation (https://www.csrkt.org.cn), the total number of kidney transplants in mainland China was only 12,712 in the same year, highlighting a challenge posed by organ shortages. Consequently, considerable efforts have been dedicated to expanding the donor pool, including accepting donors with syphilis. According to Chadban et al. (*3*) and the American Society of Transplantation Infectious Diseases Community of Practice (*4*), donors with syphilis might be considered after treatment, with recipient-informed consent and posttransplant prophylactic treatment.

Although several cases have documented no syphilis transmission from donors with treated syphilis (*2*,*5*,*6*), syphilis events were reported if the donors had active syphilis infection (*2*,*7*). Clinical guidelines recommend syphilis screening before donation (*3*,*4*). However, because of the urgency of organ procurement and the limited time for organ preservation, transplantation may occur before serologic testing results (*8*). Consequently, surgeons have learned only after transplantation that they had transplanted a kidney from a donor with syphilis. Moreover, large transplant centers may expand their donor eligibility criteria to encompass persons with syphilis because doing so improves transplant accessibility and substantially reduces time on the waiting list (*3*). However, even those proactive approaches increase the risk for donor-derived infection. We evaluated the risk of acquiring syphilis infection from a donated kidney among transplant pairs in western China. The study was approved by the Ethics Committee of West China Hospital, Sichuan University (2023SHEN354).

## The Study

We enrolled kidney transplantation pairs from West China Hospital, a national medical center in Sichuan, China, during 2007–2022. The reverse sequence algorithm was used for syphilis screening before donation and transplantation (Appendix Figure 1) (*9*). Donors and recipients initially underwent chemiluminescence immunoassay (CLIA, treponemal testing); if positive, testing with the toluidine red unheated serum test (TRUST, nontreponemal testing) and *Treponema pallidum* particle agglutination (TPPA, treponemal testing) were performed. Among 5,521 kidney transplants, 102 (1.8%) pairs had risk for donor-derived infection when donors tested positive for CLIA and recipients were CLIA negative (Appendix Figure 2). The 102 pairs were from western China, predominantly Sichuan Province (44.1%), followed by Chongqing (15.7%), Tibet (9.80%), Qinghai (8.8%), Gansu (5.9%), Guizhou (5.9%), Yunnan (3.9%), Guangxi (2.9%), Shanxi (2.0%), and Xinjiang (1.0%) Provinces (Appendix Figure 3, panel C). The mean age of donors was 48.7 years and recipients 33.5 years; 52.0% of donors were male and 48.0% female, and 71.6% of recipients were male and 28.4% female (Table). Living-donor kidney transplantation involved 3 pairs of spouses, 12 pairs of siblings, and 35 pairs of parent–child relationships.

**Table:** Baseline characteristics of 102 kidney transplantation pairs, western China, 2007–2022*

Characteristics	No. (%) patients				p value
	Overall, n = 102	CLIA+/TPPA–/TRUST–, n = 13	CLIA+/TPPA+/TRUST–, n = 45	CLIA+/TPPA+/TRUST+, n = 44	
Donor					
Type					0.848†
Deceased	52 (51.0)	7 (53.8)	24 (53.3)	21 (47.7)	
Living	50 (49.0)	6 (46.2)	21 (46.7)	23 (52.3)	
Living donor-recipient relationship					0.799‡
Spouses	3 (6.0)	0	1 (4.8)	2 (8.7)	
Parent-Child	35 (70.0)	4 (66.7)	14 (66.7)	17 (73.9)	
Siblings	12 (24.0)	2 (33.3)	6 (28.6)	4 (17.4)	
Sex					0.390‡
M	53 (52.0)	9 (69.2)	23 (51.1)	21 (47.7)	
F	49 (48.0)	4 (30.8)	22 (48.9)	23 (52.3)	
Mean age, y (± SD)	48.7 (8.9)	54.3 (6.2)	47.7 (6.7)	48.0 (10.8)	0.047§
Syphilis treatment of the living donor before donation					<0.001‡
Penicillin	16 (32.0)	0	13 (61.9)	3 (13.0)	
None	34 (68.0)	6 (100)	8 (38.1)	20 (87.0)	
Recipient					
Sex					0.001‡
M	73 (71.6)	4 (30.8)	37 (82.2)	32 (72.7)	
F	29 (28.4)	9 (69.2)	8 (17.8)	12 (27.3)	
Mean age, y (± SD)	33.5 (9.8)	37.7 (9.6)	32.4 (10.0)	33.5 (9.4)	0.224§
Hemodialysis					0.648‡
No	17 (16.7)	1 (7.7)	8 (17.8)	8 (18.2)	
Yes	85 (83.3)	12 (92.3)	37 (82.2)	36 (81.8)	
Peritoneal dialysis					0.892‡
No	92 (90.2)	12 (92.3)	41 (91.1)	39 (88.6)	
Yes	10 (9.8)	1 (7.7)	4 (8.9)	5 (11.4)	
Mean duration dialysis, mo, (± SD)	12.8 (16.0)	19.8 (26.8)	11.8 (14.6)	11.7 (13.0)	0.243¶
Induction therapy					0.534‡
Anti-thymocyte globulin	26 (25.5)	3 (23.1)	12 (26.7)	11 (25.0)	
Basiliximab	66 (64.7)	7 (53.8)	30 (66.7)	29 (65.9)	
None	10 (9.8)	3 (23.1)	3 (6.7)	4 (9.1)	
Maintenance immunosuppressive therapy					0.022‡
Cyclosporine	3 (2.9)	2 (15.4)	0 (0.0)	1 (2.3)	
Tacrolimus	99 (97.1)	11 (84.6)	45 (100.0)	43 (97.7)	
Antimicrobial use					0.016‡
Aztreonam	3 (2.9)	0	2 (4.4)	1 (2.3)	
Cefmetazole	47 (46.1)	7 (53.8)	23 (51.1)	17 (38.6)	
Cefoperazone	4 (3.9)	0	4 (8.9)	0	
Ceftriaxone	32 (31.4)	2 (15.4)	9 (20.0)	21 (47.7)	
Cefoxitin	2 (2.0)	0	2 (4.4)	0	
Ceftizoxime	10 (9.8)	4 (30.8)	2 (4.4)	4 (9.1)	
Imipenem–cilastatin	4 (3.9)	0	3 (6.7)	1 (2.3)	

*Persons were from Sichuan, Chongqing, Tibet, Qinghai, Gansu, Guizhou, Yunnan, Guangxi, Shanxi, and Xinjiang Province, China. CLIA, chemiluminescence immunoassay; mNGS, metagenomic next-generation sequencing; TPPA, *Treponema pallidum* particle agglutination; TRUST, toluidine red unheated serum test. †By χ^2^ test. ‡By Fisher exact test. §By Student *t*-test. ¶By Wilcoxon–Mann–Whitney U-test.

The kidney transplant pairs were divided according to donors’ serologic testing results: 13 were CLIA+/TPPA–/TRUST–, 45 were CLIA+/TPPA+/TRUST–, and 44 were CLIA+/TPPA+/TRUST+. More male than female recipients (69/89 [77.5%]) received kidneys from donors with confirmed syphilis (p = 0.001). More living donors in the CLIA+/TPPA+/TRUST– group received penicillin treatment before donation (13/21 [61.9%]) (p<0.001) compared with the other 2 groups. A total of 47 (46.1%) recipients received cefmetazole, followed by ceftriaxone (32 [31.4%]), ceftizoxime (10 [9.8%]), and aztreonam (3 [2.9%]). More recipients in the CLIA+/TPPA+/TRUST+ group received ceftriaxone (21/44 [47.7%]; p = 0.016) compared with the other 2 groups.

**Figure 1:**
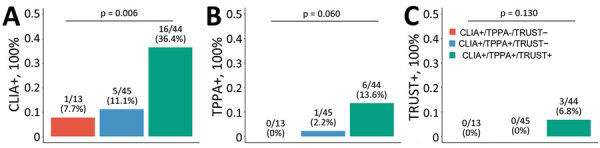
Incidence of donor-derived syphilis, China, 2007–2022. Percentage of CLIA+ (A), TPPA+ (B), and TRUST+ (C) after transplantation were determined by χ^2^ or Fisher exact test, as appropriate. CLIA+, positive by chemiluminescence immunoassay; TPPA+, positive by *Treponema pallidum* particle agglutination test; TRUST+, positive by toluidine red unheated serum test.

After transplantation, 7 recipients had confirmed syphilis (1 in the CLIA+/TPPA+/TRUST– group; 6 in the CLIA+/TPPA+/TRUST+ group), suggesting a rare risk for donor-derived syphilis (7/5521 [0.1%]). Incidence of CLIA+ was higher for persons in the CLIA+/TPPA+/TRUST+ group (16/44 [36.4%]) than in the CLIA+/TPPA+/TRUST– (5/45 [11.1%]) and CLIA+/TPPA–/TRUST– (1/13 [7.7%]) groups (p = 0.006). Incidence of TPPA+ was marginally higher for persons in the CLIA+/TPPA+/TRUST+ group (6/44 [13.6%]), than in the CLIA+/TPPA+/TRUST– (1/45 [2.2%]), and CLIA+/TPPA–/TRUST– (0/13 [0%]) groups (p = 0.060). Incidence of TRUST+ was numerically higher for persons in the CLIA+/TPPA+/TRUST+ group (3/44 [6.8%]) than in the CLIA+/TPPA+/TRUST– (0/45 [0%]) and CLIA+/TPPA+/TRUST+ (0/13 [0%]) groups (p = 0.130) (Figure 1). Specifically, 2 recipients received kidneys from CLIA+/TPPA+/TRUST+ spouses, and 1 was infected after transplantation despite the use of aztreonam. The three groups had similar kidney function, graft, and patient survival rates (Appendix Figure 4).

**Figure 2:**
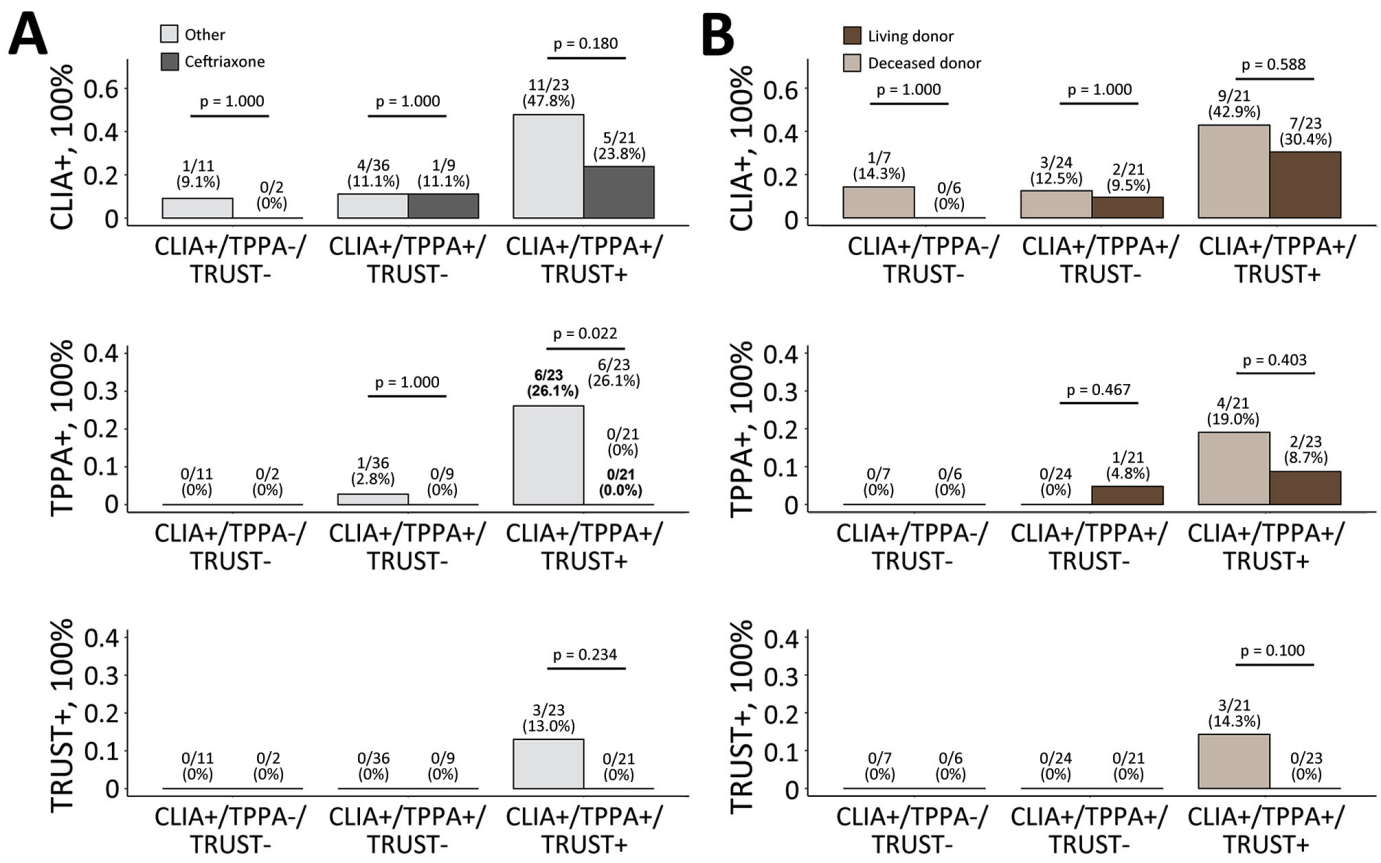
Subgroup analyses of the incidence of donor-derived syphilis, China, 2007–2022, determined by χ^2^ or Fisher exact test, as appropriate. A) Percentage of CLIA+, TPPA+, and TRUST+ after transplantation based on the use of ceftriaxone versus other antimicrobial drugs. B) Percentage of CLIA+, TPPA+, and TRUST+ after transplantation based on donor type. CLIA+, positive by chemiluminescence immunoassay; TPPA+, positive by *Treponema pallidum* particle agglutination test; TRUST+, positive by toluidine red unheated serum test.

In the CLIA+/TPPA+/TRUST+ group, use of ceftriaxone was associated with a lower incidence of TPPA+ (6/23 [26.1%] with vs. 0/21 [0%] without; p = 0.022) and with a numerically lower incidence of TRUST+ (3/23 [13.0%] with vs. 0/21 [0%] without; p = 0.234) compared with no use (Figure 2, panel A). In addition, receiving kidneys from deceased donors was associated with a numerically higher incidence of TPPA+ (4/21 [19.0%] deceased donor vs. 2/23 [8.7%] living donor; p = 0.403) and a numerically higher incidence of TRUST+ (3/21 [14.3%] vs. 0/23 [0.0%]; p = 0.100) compared with receiving kidneys from living donors (Figure 2, panel B).

Through systematic searching, we identified 9 publications (1987–2023) with sample sizes of 1–28 participants (Appendix Table). Of 65 transplant pairs, 6 recipients were CLIA+ and 4 were TPPA+ after transplantation, despite differences in donor syphilis status and recipient antimicrobial prophylaxis.

## Conclusions

We detected 7 potential donor-derived syphilis infection events among >5,000 kidney transplantations. The reverse sequence algorithm we used for syphilis screening is more sensitive than the traditional algorithm for detecting early or late latent syphilis (*10*). The extremely low incidence of syphilis transmission can be attributed to several factors: donors with syphilis were uncommon, accounting for <2% in the large cohort; living donors had the opportunity to receive treatment before donation; and after transplantation, antimicrobial prophylaxis was administered to recipients. Similarly, in a large-scale cohort study enrolling 1,460 liver recipients and 3,072 kidney recipients, 6 diagnoses of syphilis were new (*11*).

*T. pallidum* can persist in various tissues and organs at different stages of infection, in untreated and treated persons (*12*). Our findings suggest that using donors with treated syphilis poses a lower risk for infection among recipients than using donors with active syphilis. Despite the relatively low transmission risk from donors with treated syphilis, our study reported 1 infected recipient who received the kidney from his mother. Although uncommon, a possible reason could be false-negative TRUST result for persons with secondary syphilis and early latent syphilis. A similar case was reported after liver transplantation, in which a recipient received a TPPA+/Venereal Disease Research Laboratory test–negative liver graft (*13*), which clinically emphasized the role of syphilis treatment before donation, particularly in living-donor kidney transplantation.

In our study, 3 recipients received kidneys from their spouses. By chance, 1 recipient was infected after receiving a kidney from his spouse with active syphilis infection even when prophylactic measures were implemented. In the nationwide cohort study evaluating the burden of sexually transmitted infections after transplantation, 25 of 3,612 recipients were confirmed to have acquired infections, including 1 case of syphilis (*14*). Those findings underscore the imperative for recipients and their spouses to undergo treatment.

A limitation of our study is its retrospective design. In addition, the number of kidney transplants from serologically positive donors was small, potentially limiting our ability to estimate the overall risk for donor-derived syphilis. Last, antimicrobial prophylaxis against syphilis infection varied in our study.

In summary, donor-derived syphilis transmission was rare after kidney transplantation. Risk was increased if the donor had active syphilis and decreased if the recipient received prophylactic ceftriaxone.

Appendix Additional information for study of risk for donor-derived syphilis after kidney transplantation, China, 2007–2022.
