# Supplementary material for: Risk for Donor-Derived Syphilis after Kidney Transplantation, China, 2007–2022
Source: Emerg Infect Dis. 2024 Jul;30(7):1420–4. doi: 10.3201/eid3007.240009 (PMC11210647; doi:10.3201/eid3007.240009)
Supplement: Appendix — Additional information for study of risk for donor-derived syphilis after kidney transplantation, China, 2007–2022. [file 24-0009-Techapp-s1.pdf]

Article DOI: https://doi.org/10.3201/eid3007.240009

EID cannot ensure accessibility for supplementary materials supplied by authors. Readers who have difficulty accessing supplementary content should contact the authors for assistance.

Risk for Donor-Derived Syphilis after Kidney Transplantation, China, 2007–2022

Appendix

Methods

Patient selection

Exclusion criteria were applied based on donor and recipient characteristics. Donors were excluded if they met any of the following criteria: (1) missing CLIA data; (2) CLIA-; (3) CLIA+ but no TPPA or TRUST data. Recipients were excluded if they met any of the following criteria: (5) CLIA+; (6) lost during follow-up.

Statistical Analyses

Statistical analyses were performed using R software (Version 4.0.0). Statistical significance was set at $P < 0.05$. Qualitative data are presented as counts (percentages), and quantitative data are presented as mean and standard error (SD) or median and interquartile range (IQR). Comparisons between groups were performed using the chi-square test or Fisher's exact test for categorical variables, Student's t-test was used for continuous variables with normal distribution, and the Wilcoxon–Mann–Whitney U-test was used for continuous variables with non-normal distribution. We used Kaplan–Meier survival curves with log-rank tests to estimate and compare graft and patient survival.

References

1. Fernández García OA, Singh AE, Gratrix J, Smyczek P, Doucette K. Serologic follow-up of solid organ transplant recipients who received organs from donors with reactive syphilis tests: A retrospective cohort study. Clin Transplant. 2023;37:e14896. PubMed https://doi.org/10.1111/ctr.14896

2. Laowalert S, Mavichak V, Boonnim K, Naitook N, Aekkachaipitak N, Watcharananan S. Prevalence and outcome of kidney transplantation from organ donor infected with syphilis. Transplantation. 2020;104(suppl 3):S330. https://doi.org/10.1097/01.tp.0000700188.91951.13

3. Morrison M, Hurtik M, Lvon GM, Pouch S. Syphilis transmission in solid organ transplantation. Am J Transplant. 2019;19(suppl 3):723.

4. Tariciotti L, Das I, Dori L, Perera MTPR, Bramhall SR. Asymptomatic transmission of *Treponema pallidum* (syphilis) through deceased donor liver transplantation. Transpl Infect Dis. 2012;14:321–5. PubMed https://doi.org/10.1111/j.1399-3062.2012.00745.x

5. Marek A, Inkster T. A syphilis-positive organ donor — management of the cardiac transplant recipient: a case report and review of the literature. Sex Transm Dis. 2012;39:485–6. PubMed https://doi.org/10.1097/OLQ.0b013e318249db35

6. Cortes NJ, Afzali B, MacLean D, Goldsmith DJ, O'Sullivan H, Bingham J, et al. Transmission of syphilis by solid organ transplantation. Am J Transplant. 2006;6:2497–9. PubMed https://doi.org/10.1111/j.1600-6143.2006.01461.x

7. Ko WJ, Chu SH, Lee YH, Lee PH, Lee CJ, Chao SH, et al. Successful prevention of syphilis transmission from a multiple organ donor with serological evidence of syphilis. Transplant Proc. 1998;30:3667–8. PubMed https://doi.org/10.1016/S0041-1345(98)01185-3

8. Caballero F, Domingo P, Rabella N, López-Navidad A. Successful transplantation of organs retrieved from a donor with syphilis. Transplantation. 1998;65:598–9. PubMed https://doi.org/10.1097/00007890-199802270-00029

9. Gibel LJ, Sterling W, Hoy W, Harford A. Is serological evidence of infection with syphilis a contraindication to kidney donation? Case report and review of the literature. J Urol. 1987;138:1226–7. PubMed https://doi.org/10.1016/S0022-5347(17)43558-0

Appendix Table. Published literature utilizing donors with syphilis infection in solid organ transplantation

Reference	Transplantation type	Period	Donor information	Donor treatment	Recipient therapy	CLIA/ELISA positive	TPPA/TPHA positive	TRUST/RPR/VDRL positive
Fernández 2023 (*1*)	Liver, kidney, lung, heart	2020-2022	11 TPPA+ donors	7 Treated; 2 unavailable; 2 untreated	15 PEN; 5 None; 4 Ceftriaxone; 1 doxycycline	3/25	0/25	0/25
Laowalert 2020 (*2*)	Kidney	1992-2019	6 TPHA+/RPR- donors; 3 TPHA+/RPR+ donors	Unavailable	All 3 recipients with RPR-positive donor were treated; Only 3 of 6 recipients with negative-RPR donor were prescribed post-exposure prophylaxis	Unavailable	0/9	0/9
Morrison 2019 (*3*)	Solid organ transplantation	2008-2018	8 RPR+/TPPA+ donors; 20 RPR+/not confirmed (no data) donors	Unavailable	9 penicillin G potassium; 19 not treated/not documented	Unavailable	1/16; 12 not tested	1/16; 12 not tested
Tariciotti 2012 (*4*)	Liver	2011	1 TPPA+/VDRL - donor	Unavailable	Doxycycline	1/1	1/1	0/1
Marek 2012 (*5*)	Heart	2011	1 TPPA+/VDRL - donor	Unavailable	Benzathine penicillin	0/1	0/1	0/1
Cortes 2006 (*6*)	Kidney	2003	1 TPPA+/RPR+ donor	Unavailable	Benzyl penicillin	2/2	2/2	0/2

Reference	Transplantation type	Period	Donor information	Donor treatment	Recipient therapy	CLIA/ELISA positive	TPPA/TPHA positive	TRUST/RPR/VDRL positive
Ko 1998 (*7*)	Liver, kidney, lung, Heart	1998	1 TPHA+/VDRL + donor	Unavailable	1 Cefamandole; 1 Clindamycin+Ceftazidime; 1 Vancomycin+Ceftazidime; 1 Sulbenicillin; 1 Cefmetazole; 1 Fosfomycin. All recipients also recived 3-week benzathine penicillin	0/6	0/6	0/6
Caballero 1998 (*8*)	Kidney, Liver, Heart	1998	1 TPHA+/RPR- donor	Unavailable	penicillin	Unavailable	0/3	0/3
Gibel 1987 (*9*)	Kidney	1987	1 fluorescent treponema antibody-positive donor	Unavailable	penicillin	Unavailable	0/2	Unavailable

TRUST: Toluidine Red Unheated Serum test; VDRL: Venereal Disease Research Laboratory; RPR: rapid plasma reagin; TPPA: treponema pallidum particle agglutination assay; CLIA: chemiluminescence immunoassay; TPHA: treponema pallidum hemagglutination assay; ELISA: enzyme-linked immunosorbent assay.

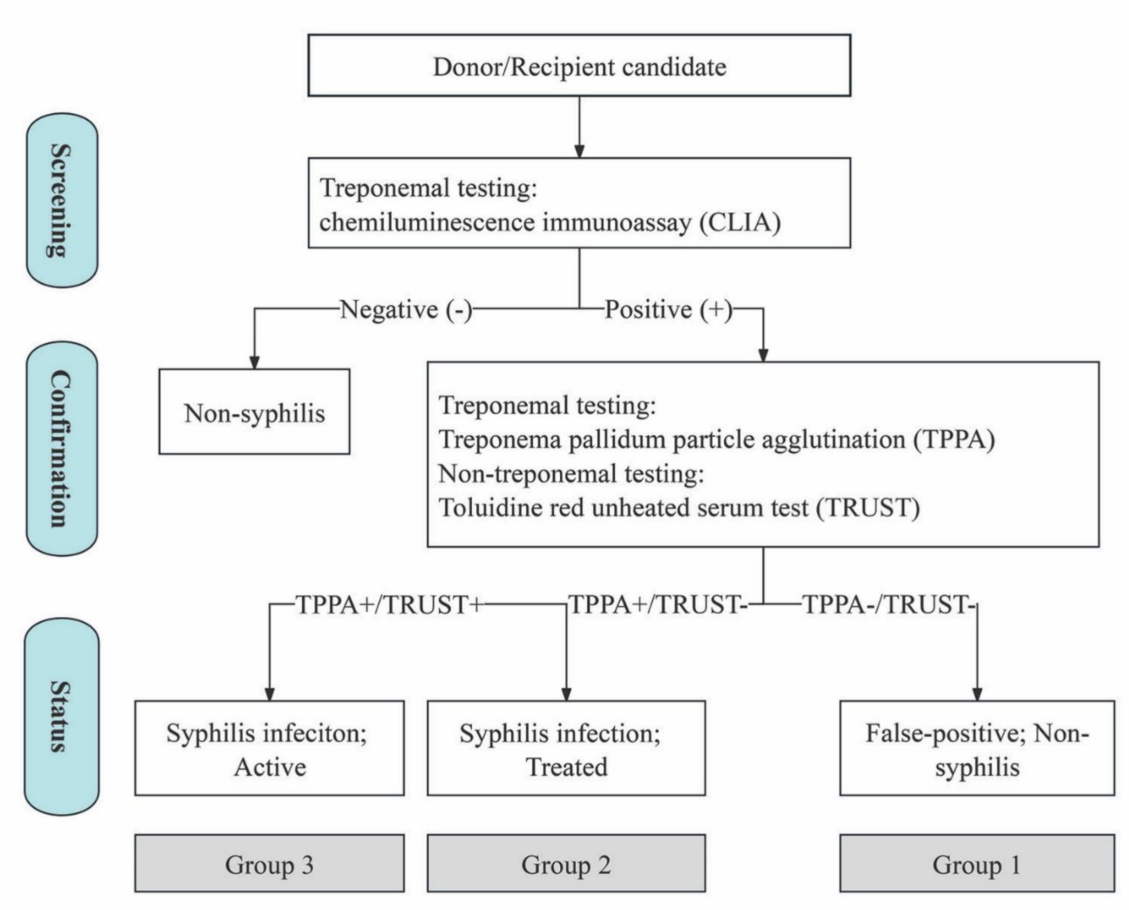

Appendix Figure 1. Reverse sequence algorithm for syphilis screening, China, 2007–2022.

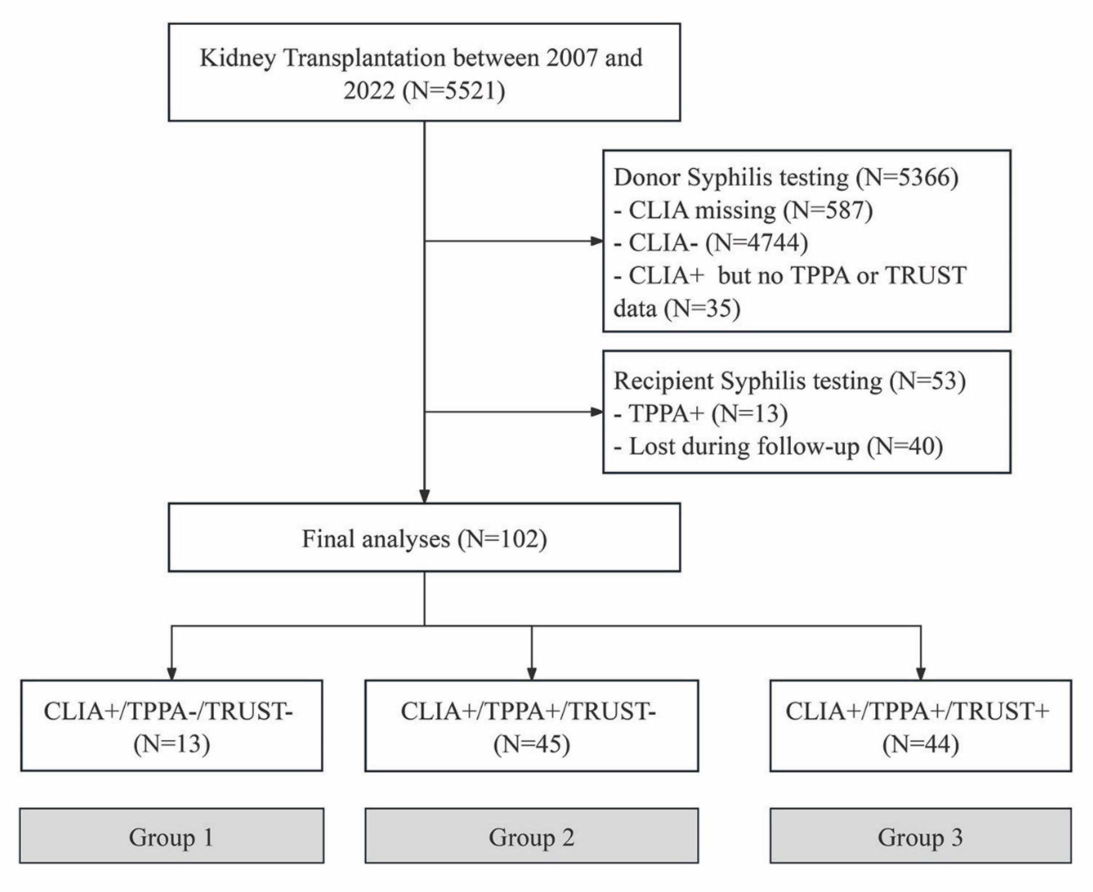

Appendix Figure 2. Patient selection, China, 2007–2022.

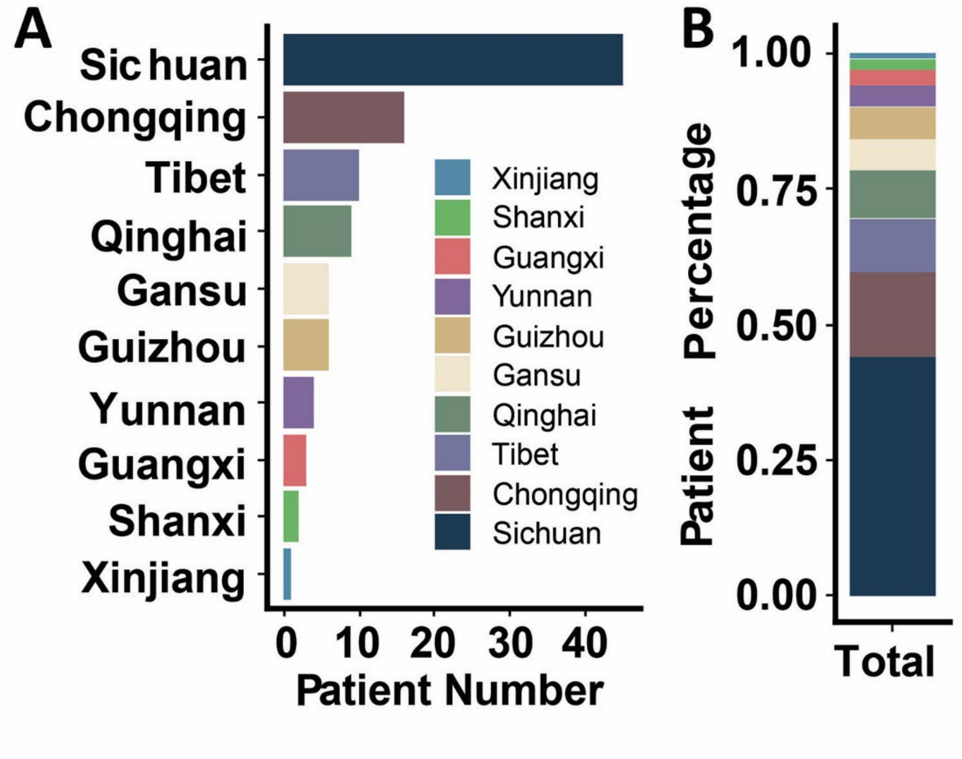

Appendix Figure 3. Patient distribution, China, 2007–2022.

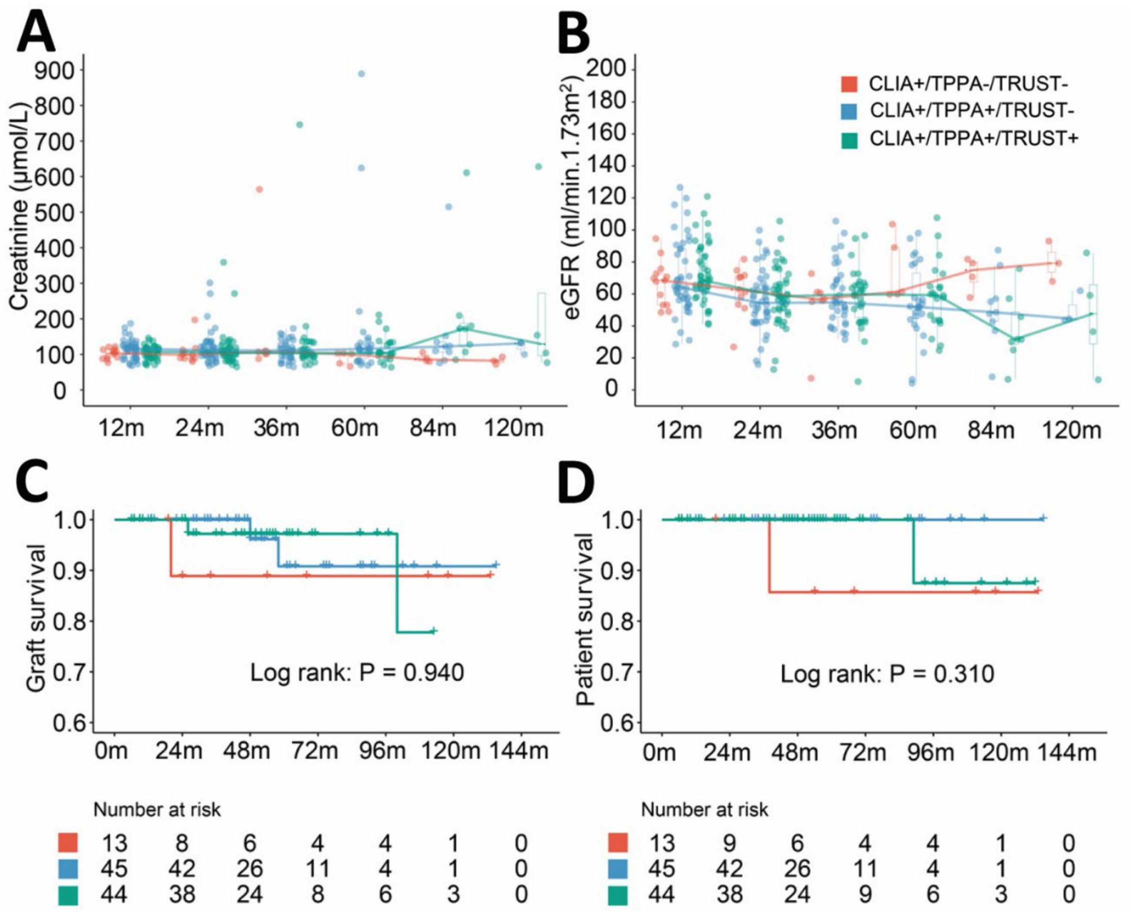

Appendix Figure 4. Clinical outcomes after kidney transplantation from donors with syphilis to serologically negative recipients, China. A) Boxplot comparing creatinine levels (μmol/L) in different time points after transplantation. Each circle represented a recipient. B) Boxplot comparing eGFR levels (ml/min/1.73m2) in different time points after transplantation. eGFR: estimated glomerular filtration rate. Each circle represented a recipient. C) Graft survival by Kaplan-Meier survival curve with log-rank test. D) Patient survival by Kaplan-Meier survival curve with log-rank test. Shading indicates 95% credibility intervals.
